# Effects of Heavy Metal Exposure from Leather Processing Plants on Serum Oxidative Stress and the Milk Fatty Acid Composition of Dairy Cows: A Preliminary Study

**DOI:** 10.3390/ani12151900

**Published:** 2022-07-26

**Authors:** Chuanyou Su, Xueyin Qu, Yanan Gao, Xuewei Zhou, Xue Yang, Nan Zheng

**Affiliations:** 1 Milk and Dairy Product Inspection Center of the Ministry of Agriculture and Rural Affairs, Institute of Animal Sciences, Chinese Academy of Agricultural Sciences, Beijing 100193, China; suchuanyou@henau.edu.cn (C.S.); quxueyin@hotmail.com (X.Q.); gyn758521@126.com (Y.G.); zhou_xuewei@126.com (X.Z.); yangxue234723@126.com (X.Y.); 2 College of Animal Science, Henan Agriculture University, Zhengzhou 450000, China; 3 Tianjin Mengde Group Co., Ltd., Tianjin 300400, China

**Keywords:** cow, milk fatty acid, heavy metals, oxidative stress

## Abstract

**Simple Summary:**

The leather industry has raised the blood lead and arsenic levels of cows in nearby farms. Significant decreases in serum GST, GPX, and GSH activity were observed in the polluted area. Milk linoleic acid (C18:2n6c) was significantly reduced in the polluted area. Heavy metal exposure through leather industry imposes oxidative stress on cows, leading to modifications in the unsaturated fatty acids of milk.

**Abstract:**

This study investigated whether unsaturated fatty acids in milk and the oxidative status of cows are affected by heavy metal exposure due to leather processing. The blood lead (Pb) concentrations in cows from two farms in the polluted area were 16.27 ± 8.63 μg/L, respectively, which were significantly (*p <* 0.05) higher than the blood Pb concentrations in cows from an unpolluted farm (6.25 ± 3.04 μg/L). There were significantly (*p* < 0.05) lower levels of glutathione S-transferase (GST), glutathione peroxidase (GPX), and glutathione (GSH) in the serum of cows from the polluted area compared to the levels in cows from an unpolluted area. The linoleic acid (C18:2n6c) content in milk from the polluted area was 15% lower than in the control area. There was a significant correlation between linoleic acid in milk with the blood Pb and serum GSH levels. Heavy metals can alter fatty acid synthesis through oxidative stress, which may be the mechanism by which heavy metals affect fatty acid synthesis in milk.

## 1. Introduction

There are about 3850 leather and leather-product processing plants in China [1]. Leather processing is an important source of heavy metal pollution. The industry-fodder-animal pathway is considered to be a major source of animal exposure to metals [2]. In our previous study, heavy metal concentrations in pasture soil, water, and silage in the vicinity of a factory were found to be higher than in a control area, which led to high levels of heavy metals in milk [3].

Heavy metals are transported to various organs in the body through blood circulation, causing damage to tissues and organs [4]. One consequence of environmental pollution is a high heavy metal load on animals, which can leave their health in a sub-optimal condition. Lipid peroxidation is considered to be the initial reaction in heavy metal toxicity [5]. Metal intoxication promoted the generation of reactive oxygen species (ROS), which either triggered protective mechanisms or caused oxidative cellular damage, such as lipid peroxidation [6]. Many organisms have developed antioxidant defense systems that help them to cope with ROS. The antioxidant defense system consists of both enzymatic and non-enzymatic components [7].

To minimize the negative effects of ROS, animals have evolved effective antioxidant defenses, which includes the enzymes catalase (CAT), glutathione peroxidase (GPX), glutathione reductase (GR), and also the multifunctional enzyme glutathione S-transferase (GST) [8,9,10]. For this reason, the evaluation of antioxidant responses and lipid peroxidation levels has been extensively used to assess the effects of metal exposure in pigs [11], aquatic organisms [12,13,14,15], humans [16], and rats [17].

Animal blood can be a good indicator of environmental heavy metal contamination [18,19]. Previous studies have reported high blood Pb levels in animals around industrial and contaminated areas [20,21].

Fatty acids can be useful biomarkers in the determination of the role of the altered synthesis of eicosanoids in the mechanisms of altered bone metabolism associated with abnormal exposures to lead (Pb) and other heavy metals [22]. Several studies have shown a relationship between elevated tissue Pb and both oxidative stress biomarkers and fatty acid composition [23,24,25,26]. Lead exposure has been shown to alter the ω-3 polyunsaturated fatty acids (PUFAs) and ω-6 PUFAs composition of milk in rats [27].

Milk is considered a “nearly complete food”, especially for infants, children, and elderly people [28]. It is a good source of fatty acids [29,30]. Whether exposure to heavy metals through leather processing can increase lipid peroxidation in cows and change the unsaturated fatty acid composition in raw milk has not been reported. This study measured the heavy metal concentrations in cow blood in an area affected by the leather industry. It identified the adverse effects of heavy metal pollution on oxidative stress in serum and the fatty acid profile in the milk of cows. The links between blood heavy metal levels, serum oxidative stress biomarkers, and the milk fatty acid profile were determined.

## 2. Materials and Methods

### 2.1. Study Area

The leather industry is the largest pillar industry in Wuji County, Shijiazhuang, China, and the output of the local leather industry accounts for 10% of the national total [31]. The details of the study area are reported in Su et al. [3]. In brief, samples were obtained from healthy cows, raised in three farms that operated a mid-level extensive agricultural production system in Shijiazhuang, Hebei Province, China, with varying proximity to industrial activities. Farms A and B were located in the vicinity of leather processing plants (within 10 km of the source of pollution) in Wuji County. Farm C (control) was located far from (>50 km) the leather processing plants and was considered to be a non-polluted area that served as a control [3]. The three farms provided raw milk to the same dairy-processing corporation. The cows were fed by house feeding. Silage was sourced from local areas near the individual farms. The same fodder, except silage, was used for the cows at all three farms. A total of 45 Holstein dairy cows (2.49 ± 0.66 parity, 15 cows per farm) were selected. The mean days in milk (DIM) of the cows selected were 197.3 ± 15.9 days. The mean milk yield for cows was 24.20 ± 3.14 kg.

### 2.2. Sampling

Blood and serum samples were obtained from cows at 5:30 a.m., which was before the morning feed. Blood was sampled from the right jugular vein into vacutainer tubes and stored at 4 °C for a later heavy metal analysis [32]. Serum samples were obtained according to a previous method [33]. Briefly, blood samples (5 mL) were collected in pro-coagulation tubes and then centrifuged at 3000× *g* at 4 °C for 15 min. Serum was separated and packed in Eppendorf tubes that were stored at −80 °C.

Milk samples were collected from individual cows during the milking procedure at 06:00 and 17:00 and were mixed in the proportion of 6:4. After sampling, raw milk was stored in 200 mL polyethylene plastic bottles at −20 °C. This study was approved by the ethics committee of the Chinese Academy of Agricultural Sciences (Ethics Approval No. SL2016-023).

### 2.3. Measurement of Heavy Metals

Blood heavy metals were measured by inductively coupled plasma mass spectrometry (ICP-MS) after digestion with HNO_3_ and H_2_O_2_. Briefly, 1 mL blood was added followed by 5 mL of HNO_3_ (65%, Suprapur, Merck, Darmstadt, Germany) and 2 mL of H_2_O_2_ (30%, Suprapur). The mixture was digested in a microwave-assisted reaction system (CEM MARs 6, Matthews, NC, USA) according to a previously reported program [32]. After cooling to room temperature, the mixture was then diluted to 50 mL with ultrapure water. After filtration through a membrane (0.22 μm), the solution was analyzed by inductively coupled plasma mass spectrometry (7700 Series ICP-MS; Agilent Technologies, Santa Clara, CA, USA). Standard calibrations (five points) were developed for each metal. The correlation coefficients were >0.999 before determinations were made.

### 2.4. Oxidative Stress Analysis

The serum malondialdehyde (MDA), superoxide dismutase (SOD), GST, GSH, GPX. GR, and CAT were determined using ELISA kits (Nanjing Jiancheng Bioengineering institute, China). Briefly, a 20 μL sample, 20 μL enzyme working fluid, and 200 μL substrate-applied solution were added. The mixture was then incubated for 20 min at 37 °C. The SOD levels were measured at 450 nm using a microplate reader. For GST, 50 μL samples were mixed with 200 μL reagent I and then centrifuged at 3500 rpm for 10 min. After settling for 5 min, the light absorbance of the supernatant was measured at 405 nm with a microplate reader.

For the GSX enzymatic reaction, 0.2 mL 1 mmol/L GSH in a non-enzyme tube was placed in a water bath for 5 min at 37 °C, and then 0.1 mL reagent I was added. The mixture was placed back into the water bath for 5 min at 37 °C. Then, 2 mL reagent II and 0.2 mL of sample were added. The mixture was centrifuged at 3500 rpm for 10 min. Then, 0.2 mL 1 mmol/L GSH and 0.2 mL sample were placed into an enzyme tube, which was placed in a water bath for 5 min at 37 °C. Then, 0.1 mL reagent I was added. The mixture was placed back into the water bath for 5 min at 37 °C, and then, 2 mL reagent II was added. The mixture was centrifuged at 3500 rpm for 10 min. For the color reaction, 1 mL supernatant, 1 mL reagent III, 0.25 mL reagent 4, and 0.05 mL reagent 5 were mixed. After sitting for 15 min, the OD value was measured at 412 nm.

For the CAT enzymatic reaction, reagent I and reagent II were preheated at 37 °C. Then, 0.1 mL serum, 1 mL reagent I, and 0.1 mL reagent II were mixed and then reacted for 60 s at 37 °C. Then, 1 mL reagent II and 0.1 mL reagent III were added and mixed well. Light absorbance was measured at 405 nm with a diameter of 0.5 cm.

For the GSH enzymatic reaction, 0.5 mL sample and 0.2 mL reagent I were mixed and then centrifuged at 3500 rpm for 10 min. Then, 100 μL of the supernatant was mixed with 100 μL reagent II and 25 μL reagent III. After sitting for 5 min, the light absorbance was measured at 405 nm using a microplate reader.

For the MDA enzymatic reaction, 0.1 mL serum and 0.1 mL reagent I were mixed and shaken, and then 3 mL reagent II and 1 mL reagent III were added. The tube was mixed by vortexing and then placed in a water bath for 40 min at 90 °C. Then, the tube was centrifuged at 3500 rpm for 10 min. The light absorbance of the supernatant was measured at 532 nm, with a diameter of 1 cm.

For the GR enzymatic reaction, 50 μL serum and 2.4 mL working solution were mixed and shaken. Light absorbance was measured at 340 nm. The light absorbance was measured again at 340 nm after the tube was placed in a water bath for 2 min at 37 °C.

For the metallothionein (MT) enzymatic reaction, 50 μL of the diluted standard was placed into a reaction well together with 50 μL of serum. Then 50 μL of the antibody was added immediately. The membrane plate was covered, gently shaken, and incubated at 37 °C for 1 h. The liquid in the holes was shaken off, and each hole was filled with washing liquid. The plate was shaken for 30 s to remove the washing liquid and patted dry with absorbent paper. This operation was repeated three times. Then, 80 μL of the affinity chain enzyme horseradish peroxidase (HRP) was added to each well. The plate was gently shaken and incubated at 37 °C for 30 min. The liquid in the hole was shaken off, and each hole was filled with washing liquid. The plate was shaken for 30 s to remove the washing liquid and patted dry with absorbent paper. This operation was repeated three times. Then, 50 μL substrate A and 50 μL substrate B were added to each well. The plate was gently shaken and incubated at 37 °C for 10 min, avoiding light. The enzyme label plate was then removed, 50 μL termination solution was immediately added, and the optical density (OD) was determined at 450 nm.

### 2.5. Determination of Fatty Acids

Fatty acids were determined by the method developed by Wang et al. (2011) [34]. Briefly, the milk sample was fully mixed after thawing in a cold water bath. A 2 mL milk sample was added to a 4 mL n-hexane/isopropanol mixed solution, after blending with 2 mL sodium sulfate solution. The mixture was centrifuged at 5300 rpm for 20 min at room temperature. The supernatant was placed in a 20 mL hydrolysis tube and dried in a nitrogen flow. Then, a 2 mL mixed solution of sodium hydroxide and methanol was added. The mixture was placed in a water bath for 15 min at 50 °C. After cooling, 2 mL of a hydrochloric acid/methanol solution was added, and the mixture was placed in a water bath for 1.5 h at 80 °C. Then, 3 mL pure water and 6 mL n-hexane were added after cooling to room temperature. The mixture was allowed to stand or centrifuged for stratification. The upper liquid reached a constant volume of 10 mL and was dried by anhydrous sodium sulfate. Final measurements were made by gas chromatography-mass spectrometry (GC-MS) using an external standard to ensure a quantitative method. The gas chromatographic conditions were as follows: chromatographic column: HP-88 (100 m × 0.25 mm × 0.25 μm); column temperature: 120 °C for 10 min, then increased to 230 °C at 3.2 °C /min and maintained for 35 min; inlet temperature of 250 °C; detector temperature of 300 °C; carrier gas was nitrogen; constant pressure of 190 kPa; split ratio of 1:50; and sample size of 2 μL.

### 2.6. Data Analysis

The data were analyzed with SPSS (IBM, Endicott, NY, USA) version 20. Results were expressed as a mean ± standard deviation (SD). The data were compared using a non-parametric Kruskal-Wallis test. Differences were considered to be statistically significant at *p <* 0.05. To explore the relationships, a Spearman’s correlation analysis was conducted.

## 3. Results and Discussion

### 3.1. Heavy Metal Residues in Blood

Animal blood can be a good indicator of environmental heavy metal contamination [18,19]. Blood is the tissue used most frequently to estimate exposure to heavy metals and its association with health outcomes [32,35]. The mean arsenic (As), Pb, and chromium (Cr) concentrations in the blood of cows grazing close to leather processing plants were higher than the levels in cows from the control area, which might be due to the ingestion of contaminated forage. Animal feed is the main source of heavy metals [2,36]. In our previous study, the heavy metals in silage and the total mixed ration (TMR) of farms near a leather processing area were found to be higher than those in the control group [3].

The blood As levels of cows from farms in the polluted area were 1.61 ± 0.28 μg/L, which were significantly (*p <* 0.05) higher than the levels in cows from an unpolluted area (1.25 ± 0.18 μg/L). The blood Pb levels of cows from farms in the polluted area were 16.27 ± 8.63 μg/L, which were significantly (*p <* 0.05) higher than the levels in cows from an unpolluted area (6.25 ± 3.04 μg/L). The blood Cr levels in cows from farms in the polluted area were 2.54 ± 1.51 μg/L, which was higher than the levels in cows from an unpolluted area (1.55 ± 0.56 μg/L) (Table 1). There was no significant difference in the cadmium (Cd) levels between the control and polluted areas.

Heavy metals in the blood of cows reared in the polluted area increased. The blood Pb concentration in cows reared in irrigated areas with wastewater was 15 ± 4 μg/L [37]. The blood Pb concentration in cattle living near trunk roads was 193.3 ± 95.35 μg/L [38]. The blood Pb concentration in cattle from a Pb-polluted mining area in Spain was 56.5–805.1 μg/L [39]. These levels were higher than those detected in the present study. The blood Pb concentrations recorded in the present study were higher than those in blood (ND–34.0 μg/L) [40] and plasma (0.659 ± 0.146 μg/L) [41] reported in Spain. The As contents were within the range reported previously for cows reared in areas irrigated with wastewater [37] and in Galicia, Spain [40]. The blood Cd concentrations in this study were within the range (ND–1.65 μg/L) of cows reared in Galicia, Spain [40].

### 3.2. Effect of Heavy Metal Exposure on Oxidative Stress in Serum

**Figure 1:**
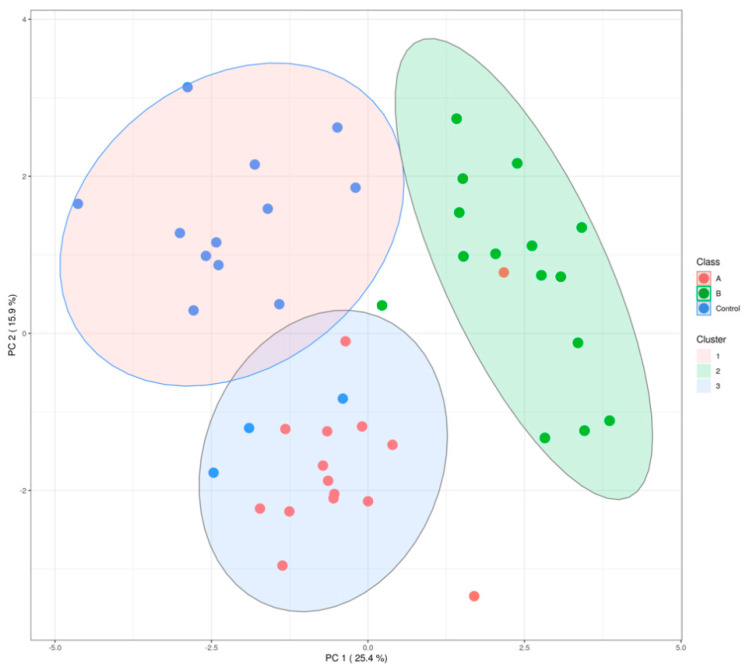
The PCA analysis of heavy metals in blood and oxidative stress. Note: A, Farm A; B, Farm B; Farm A and Farm B are polluted farms; Control, Farm C, unpolluted farm.

The distribution of metals in blood, and oxidative stress levels in the serum of cows were investigated using a principal component analysis (PCA). The PCA results (Figure 1) showed a clear separation between the control (C), and polluted areas (farms A and B). This could be attributed to the differences in heavy metal stress in the cows in the different areas.

Heavy metals, especially Pb, are environmental toxicants that can induce oxidative stress by stimulating the excess generation of ROS, which has been reported to be an important mechanism underlying Pb toxicity [42]. The ability of Pb to cause oxidative stress in blood has been suggested to be the underlying molecular mechanism of some Pb-related pathologies. Metal intoxication can accelerate the production of cellular ROS, which is expected to instigate a response from the antioxidant system [43]. Cellular ROS can result in damage to the normal oxidative metabolism [5]. The evaluation of lipid peroxidation levels has been used as a measure of oxidative stress induced by pollutants, including metals, as previously reported in Japanese quail following chronic Pb exposure [42], the brown mussel *Perna perna* following exposure to heavy metals [44], *Channa punctatus* in a heavy metal polluted canal [13], and wild ungulates in a Pb polluted mining area [45]. The response to oxidative stress involves key antioxidant and ancillary enzymes, such as GST, GPX, and glutathione (GSH).

Table 2 presents a summary of the analysis procedure used to determine the levels of GST, GPX, SOD, GR, CAT, MT, MDA in the serum of cows. There were significantly (*p <* 0.05) lower GST, GPX, and GSH levels in the serum of cows from polluted areas compared to the levels in cows from the unpolluted area. The GST activity was significantly (*p <* 0.05) lower (52.09 ± 21.16 U/mL) in the serum of cows from the polluted area compared to cows from the unpolluted area (68.81 ± 16.66 U/mL). The GPX activity (435.29 ± 19.14 U/L) in the polluted area was markedly lower than in the control area (501.04 ± 58.62 U/mL). The GSH activity was significantly different in cows from the polluted area (13.18 ± 7.99 μmol/L) compared to cows from the unpolluted area (33.18 ± 19.91 μmol/L), with a difference of 60.3%. The CAT and MT activity were lower in the polluted area compared to the unpolluted area (*p >* 0.05). There was no significant difference in the GR, SOD, and MDA activity between the polluted and unpolluted areas.

As a protective agent, GSH plays an important role in detoxification processes and is the first line of defense against heavy metal toxicity [11]. In the present study, the increased lipid peroxidation as a result of heavy metal exposure was accompanied by a depletion of serum GSH in cows from the leather processing area. This GSH depletion led to the production of free radicals [11]. In the present study, significantly lower levels of GSH were observed in the blood of cows reared near leather processing plants. This could be due to GSH being a sulfhydryl-rich antioxidant, which gives GST a strong electron donating property. It donates electrons to the ROS/free radicals and is readily oxidized to glutathione disulfide (GSSG). This rapid utilization of both GST and GSH led to a decline in ROS levels [46,47].

Glutathione S-transferase activity will counteract oxidative cellular damage [48]. The decrease in GST activity in cow blood in the present study agreed with the findings in the liver and kidney tissue of *Channa punctatus* from a heavy metal polluted canal [13] and in the liver of *Notophterus notopterus* from the Mahanadi River, India, which were related to the heavy metal concentrations [14].

High levels of ROS in animal tissues are a result of their enhanced rate of generation and/or a decline in the process of cellular antioxidant defense (free radicals in biology and medicine). The first lines of antioxidant defense are enzymes [14]. Glutathione peroxidase removes H_2_O_2_ and organic peroxides by coupling their reduction to the oxidation of GSH. Mohanty et al. [14] observed a significant reduction in GPX activity in the liver of *Notopterus notopterus* from the Mahanadi River as a function of the heavy metal concentration. In our study, the serum GPX of cows reared in polluted areas was significantly lower than in the serum GPX of cows from the control area.

### 3.3. Changes in the Fatty Acid Content in Raw Milk from Polluted Areas

Cow milk is an important source of dietary lipids and contains an abundance of bioactive fatty acids. The fatty acid content was observed to be significantly lower in the milk of cows from polluted areas compared to the control area (Table 3). In particular, the proportional content of linoleic acid (c18:2n6c) decreased. This was an important observation because C18:2n6c has a beneficial effect on human health, improving the sensitivity to insulin, and thus reducing the incidence of type 2 diabetes [49].

**Figure 2:**
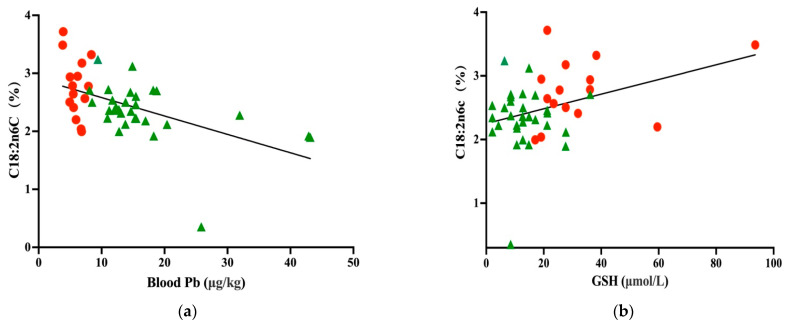
Relationship between the C18:2n6c content in milk and (**a**) the blood Pb concentration and (**b**) serum GSH level. 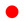 Unpolluted area 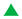 polluted area.

There was a significant correlation between the C18:2n6c content in raw milk and both the blood Pb level in cows (n = 45, r = −0.536, *p <* 0.01) and serum GSH (n = 45, r = 0.381, *p* < 0.01) (Figure 2).

Lipids are particularly susceptible to free radical damage and the biomarkers of lipid peroxidation are considered to be the best indicators of oxidative stress [50]. Polyunsaturated fatty acids are susceptible to degradation by oxygen, heat, and light, and their presence increases the potential for lipid peroxidation [51]. The decrease in the C18:2n6c content in polluted areas may be secondary to the impact of lipid peroxidation caused by heavy metals [23,52].Heavy metals alter fatty acid synthesis through oxidative stress [23], which may be the process by which heavy metals affect fatty acid synthesis in milk [27].

Lipids, in particular those that are polyunsaturated, are prone to oxidation. The decrease in the C18:2n6c content of milk may be related to the oxidation of unsaturated fatty acids.

It was found that the percentage of C18:2n6c in milk was related to the heavy metal concentration in blood. It was also related to antioxidant indexes. Heavy metals can alter unsaturated fatty acid synthesis through oxidative stress, which may be the mechanism by which heavy metals affect fatty acid synthesis in milk.

## 4. Conclusions

The leather industry has had a substantial impact on the blood Pb and As levels of cows on nearby farms. Significant decreases in serum GST, GPX, and GSH activity were observed in the polluted area. Linoleic acid (C18:2n6c) was significantly altered in the polluted area. Heavy metal exposure imposes oxidative stress on cows, leading to modifications in the unsaturated fatty acids of milk.

## Figures and Tables

**Table 1:** Blood heavy metal levels in cows from polluted and unpolluted areas.

Metals (μg/L)	Unpolluted (n = 15)		Polluted (n = 15)		*p*
	Mean ± SD	Range	Mean ± SD	Range	
As	1.25 ± 0.18	1.04–1.66	1.61±0.28	1.19−2.18	0.000 **
Pb	6.25 ± 3.04	2.76–12.08	16.27±8.63	6.48−46.43	0.000 **
Cr	1.55 ± 0.56	1.08–2.98	2.54±1.51	1.16−9.11	0.014 *
Cd	0.125 ± 0.043	0.07–0.21	0.119±0.057	0.06−0.26	0.702

Significance levels: * *p* < 0.05, ** *p* < 0.01.

**Table 2:** Antioxidant enzymes and lipid peroxidation in the serum of cows from polluted and unpolluted areas.

Parameters	Unpolluted (n = 15)	Polluted (n = 30)
SOD U/mL	66.90 ± 8.25	69.10 ± 7.99
GST U/mL	68.81 ± 16.66 ^a^	52.09 ± 21.16 ^b^
GPX U/L	501.04 ± 58.62 ^a^	435.29 ± 19.14 ^b^
GR ng/mL	267.96 ± 114.50	292.83 ± 134.76
CAT ng/mL	23.98 ± 6.93	21.67 ± 5.66
GSH μmol/L	33.18 ± 19.91 ^a^	13.18 ± 7.99 ^b^
MT ng/mL	1458.19 ± 520.18	1195.85 ± 426.03
MDA nmol/mL	28.11 ± 19.11	20.23 ± 20.47

Note: In the same row, different letters indicate a significant difference at *p <* 0.05, which is also the case in the tables below.

**Table 3:** The fatty acid content in raw milk from polluted and unpolluted areas.

Fatty Acids (%)	Unpolluted (n = 15)	Polluted (n = 30)
C6:0	1.96 ± 0.22	1.91 ± 0.55
C8:0	1.4 ± 0.19	1.32 ± 0.52
C10:0	8.5 ± 1.52 ^a^	7.28 ± 2.19 ^b^
C12:0	4.11 ± 0.71 ^a^	3.26 ± 0.81 ^b^
C13:0	0.04 ± 0.06	0.05 ± 0.05
C14:0	12.25 ± 1.26 ^a^	11.19 ± 1.39 ^b^
C15:0	1.23 ± 0.14 ^a^	1.10 ± 0.18 ^b^
C16:0	32.01 ± 2.74 ^a^	35.72 ± 4.54 ^b^
C17:0	0.82 ± 0.22	0.75 ± 0.091
C18:0	11.13 ± 1.6	10.69 ± 2.08
C20:0	0.09 ± 0.08	0.10 ± 0.07
C23:0	0.19 ± 0.11	0.18 ± 0.07
Total saturated fatty acids	75.98 ± 8.66	75.72 ± 9.41
C14:1	1.30 ± 0.22	1.21 ± 0.31
C15:1	0.25 ± 0.17	0.21 ± 0.10
C16:1	1.58 ± 0.41	1.64 ± 0.66
C17:1	0.16 ± 0.12	0.15 ± 0.09
C18:1n9t	0.45 ± 0.24	0.45 ± 0.29
C18:1n9c	20.97 ± 2.48	21.03 ± 1.63
C20:1	0.22 ± 0.20	0.15 ± 0.11
The monoene fatty acids	24.93 ± 7.02	24.83 ± 7.20
C18:2n6t	0.29 ± 0.11	0.23 ± 0.11
C18:2n6c	2.77 ± 0.51 ^a^	2.35 ± 0.49 ^b^
C18:3n6	0.19 ± 0.19	0.19 ± 0.15
C18:3n3	0.35 ± 0.22	0.29 ± 0.13
C20:3n3	0.23 ± 0.19	0.25 ± 0.19
Total poly unsaturated fatty acids	3.83 ± 1.05	3.31 ± 0.89

Note: Superscript lower-case letters (a, b) different in the same row indicate significant differences (*p* < 0.05).

## Data Availability

No new data were created or analyzed in this study. Data sharing is not applicable to this article.
